# Contributions of the oligopeptide permeases in multistep of *Vibrio alginolyticus* pathogenesis

**DOI:** 10.1002/mbo3.511

**Published:** 2017-07-17

**Authors:** Wenjia Liu, Lixing Huang, Yongquan Su, Yingxue Qin, Lingmin Zhao, Qingpi Yan

**Affiliations:** ^1^ Key Laboratory of Healthy Mariculture for the East China Sea Fisheries College Ministry of Agriculture Jimei University Xiamen China; ^2^ State Key Laboratory of Large Yellow Croaker Breeding Ningde China; ^3^ College of Ocean & Earth Sciences Xiamen University Xiamen China

**Keywords:** adhesion, biofilm, hemolytic activity, Opp system, pathogenesis, RNAi, *Vibrio alginolyticus*

## Abstract

*Vibrio alginolyticus* has been associated with several diseases of cultivated marine animals, and has led to considerable economic losses. The oligopeptide permease (Opp) has been proven to play a variety of important roles in nutrition and virulence in several bacteria. In our previous research, the *opp* gene cluster was identified in *Vibrio alginolyticus* with transcriptome sequence, which also indicated that the Opp system might play roles in the regulation of adhesion. In this study, the relationship between *V. alginolyticus* virulence and the *opp* gene cluster was determined using gene silencing followed by RT‐qPCR, in vitro adhesion assay, growth curves detection in the presence of glutathione (GSH) as a toxic substrate, hemolysis assay, biofilm assay, and artificial infection. Silencing these genes led to deficiencies in adhesion, peptide internalization, biofilm production, hemolytic activity, and virulence. The expression levels of *hapr*,* hapa*,* tlh,* and *hlya*, which are important genes closely related to the hemolytic activity of *Vibrio*, were significantly downregulated in all of the RNAi groups. Furthermore, the expression of *oppA*,* oppB*,* oppC*,* oppD,* and *oppF* was significantly influenced by temperature, starvation, and pH. These results indicate that (1) *oppABCDF* contributed in multistep of *V. alginolyticus* pathogenesis, including adhesion, biofilm production, and hemolytic activity; (2) *oppABCDF* was sensitive to different temperatures, changes in pH, and increased starvation time.

## 1 INTRODUCTION

Oligopeptide permeases (Opp) have been identified in several Gram‐negative and ‐positive bacteria. These transport systems are multisubunit protein complexes that belong to the family of ATP‐binding cassette transporters (Lewinson & Livnat‐Levanon, 2017). Opp importers are located in the plasma membrane, and its main function is to capture peptides from the extracellular environment to serve as sources of plasma carbon and nitrogen (Braibant & Gilot, 2000; Monnet, 2003). Typically, Opp importers comprise a complex of five proteins. The oligopeptide‐binding protein OppA is responsible for the capture of peptides from the external medium. Two integral transmembrane proteins, OppB and OppC, are responsible for the formation of the transmembrane channel through which the oligopeptides are transported to the intracellular environment. Two membrane‐bound cytoplasmic ATP‐binding proteins, OppD and OppF, are responsible for ATP hydrolysis, thus generating energy for the peptide internalization process (Braibant & Gilot, 2000; Quiocho & Ledvina, 1996). At a genetic level, the five *opp* genes encoding the transporter are usually organized in an operon, *oppABCDF* (Green, Seth, & Connell, 2000).

In addition to their nutritional role, Opp systems have also been associated with virulence in several bacterial. Studies performed on pathogenic bacteria of the genera *Staphylococcus* sp., *Streptococcus* sp., and *Mycobacterium* sp. have shown that Opp mutant strains show reduced virulence (Coulter et al., 1998; Sassetti & Rubin, 2003; Wang et al., 2005). Resent research on Vibrios also showed that Opp systems are essential for the in vitro hemolytic activity and biofilm production (Lee et al., 2004; Wu et al., 2007). The peptides captured by Opp systems can be used as signaling molecules in intercellular communication, which allows the bacteria to coordinate the expression of specific genes at a population level. The control of virulence has been linked to communication via signal peptides (Coulter et al., 1998; Detmers, Lanfermeijer, & Poolman, 2001; Lazazzera, 2001;; Samen, Gottschalk, Eikmanns, & Reinscheid, 2004). For example, the peptides could also activate a pleiotropic virulence regulon, as demonstrated in the case of *Bacillus thuringiensis* (Gominet, Slamti, Gilois, Rose, & Lereclus, 2001; Slamti & Lereclus, 2002), or by stimulating adherence of pathogenic streptococci to human cells (Cundell, Pearce, Sandros, Naughton, & Masure, 1995; Samen et al., 2004).

*Vibrio alginolyticus* is one of the most important opportunistic pathogens and has been associated with several diseases of cultivated marine animals, including those in fish and shellfish (Heo et al., 2012), shrimp (Ahmed, Rafiquzaman, Hossain, Lee, & Kong, 2016), and those associated with coral reefs (Xie et al., 2013). The large yellow croaker (*Pseudosciaena crocea*) is one of the most economically important maricultured fish species in southeast China (Wu et al., 2014). *V. alginolyticus* has caused mass mortality of cultured large yellow croakers and has led to considerable economic losses.

The pathogenic process of bacteria is generally divided into adhesion, invasion, colonization, proliferation, and production of toxins (Huang et al., 2015). Interestingly, Kong et al. (2015) found that, compared with the unstressed control, exposure of *V. alginolyticus* to Cu, Pb, Hg, and pH = 5 was found to reduce adhesion percentages to 62.59%, 60.74%, 59.35%, and 43.42%, respectively. Furthermore, through transcriptome sequence data (Kong et al., 2015), it was previously found that the opp gene cluster of *V. alginolyticus* contains five genes: oppA, oppB, oppC, oppD, and oppF. Cu, Pb, Hg, and pH = 5 treatments significantly reduced expression of oppA, oppB, oppC, oppD, and oppF (Kong et al., 2015). These indicated that the Opp system might play roles in the regulation of adhesion in *V. alginolyticus*.

In consideration of the heavy economic losses caused by *V. alginolyticus*, identification of virulence genes in *V. alginolyticus* has raised increasing attention. However, no detailed analysis of the *opp* genes of *V. alginolyticus* has been carried out. In this study, we tried to determine the relationship between *V. alginolyticus* virulence and the *opp* gene cluster.

## 2 MATERIALS AND METHODS

### 2.1 Bacterial strain and culture conditions

Pathogenic *V. alginolyticus* (ND‐01) was isolated from naturally infected *Pseudosciaene crocea* and previously identified as pathogenic by subsequent artificial infection (Kong et al., 2015; Yan, Wang, Su, & Zhang, 2001). Bacteria were preserved in physiological saline with 10% glycerol at −80°C. *V. alginolyticus* was maintained at 28°C on tryptic soy agar (TSA) supplemented with 2% NaCl and grown in Luria–Bertani (LB) broth supplemented with 2% NaCl with shaking (220 r.p.m.).

To investigate the effects of different temperatures, *V. alginolyticus* was treated according to the method of Huang, Huang, et al. (2016). Bacteria were incubated overnight in LB broth at 4, 15, 28, 37, and 44°C. After harvesting and resuspending, the bacterial suspensions were equilibrated at the same temperature (4, 15, 28, 37, and 44°C) for 30 min.

To assess the effects of different pH values, *V. alginolyticus* was prepared following the method of Huang, Huang, et al. (2016). Bacteria were incubated overnight in LB broth at different pH values (pH = 5, 6, 7, 8, and 9). The bacterial cultures were washed with phosphate‐buffered saline (PBS) (pH = 5, 6, 7, 8, and 9) (Yan, Chen, Ma, Zhuang, & Wang, 2007).

To evaluate the influence of starvation, *V. alginolyticus* was treated according to the method of Huang, Huang, et al. (2016). *V. alginolyticus* was suspended in PBS, and the bacterial suspensions were adjusted to an OD_560 nm_ of 0.3, starved at 28°C for 1, 3, 5, and 7 days. Culturable *V. alginolyticus* cells were counted using plate counting (PC) (Jiang et al., 2017; Lin et al., 2017).

All of above bacterial suspensions were adjusted to an OD_560 nm_ of 0.3 for RNA extraction and in vitro adhesion assays. Six replicates were prepared for each treatment of all the tests mentioned above.

### 2.2 Transient gene silencing

Short‐interfering RNA (siRNA) was designed according to gene sequences and synthesized by GenePharma Co. Ltd. (Shanghai, China). The negative control and treatment siRNA sequences are listed in Table S1. siRNA was electrotransferred into *V. alginolyticus* following the method described by Wang et al. (2015). After electroporation, the mixture was then incubated at 28°C for 1, 6, 12, and 24 hr prior to RNA extraction and RT‐qPCR.

### 2.3 Stable gene silencing

Stable gene silencing was performed following the method described by Darsigny et al. (2010). Five short‐hairpin RNA sequences targeting the coding regions of *oppA*,* oppB*,* oppC*,* oppD,* and *oppF* mRNAs were synthesized by Shanghai Generay Biotech Co. Ltd. (Shanghai, China) (Table S2). The annealed oligonucleotides were ligated using T4 DNA ligase (TaKaRa, Kusatsu, Japan) into the Tc operon of pACYC184 vector double digested with *Bam*HI and *Sph*I (Chang & Cohen, 1978; Qin et al., 2014). The recombinant plasmids were identified by DNA sequencing. The recombinant plasmids were transformed into *E. coli* SM10 via heat shock and then transferred from strain SM10 to *V. alginolyticus* by conjugation. An empty pACYC184 vector was used as the control. Chloramphenicol (34 μg/ml) was used to screen the stable silenced clones.

*E. coli* strain SM10 was obtained from TransGen Biotech (Beijing, China) and incubated in LB broth (220 r.p.m.) or on LB agar plates at 37°C.

### 2.4 RNA extraction and reverse transcription

Total RNA was extracted from the bacteria using TRIzol (Invitrogen, Carlsbad, CA, USA) following the manufacturer's recommended protocol. First‐strand cDNA was synthesized from the total RNA using a Revert Aid Mu‐MLV cDNA synthesis kit following the manufacturer's recommended protocol.

### 2.5 RT‐qPCR

RT‐qPCR was performed on a QuantStudio^™^ 6 Flex real‐time PCR system (ABI, Carlsbad, CA, USA) using Power SYBR Green PCR Master Mix (Applied Biosystems, Carlsbad, CA, USA). The reactions were performed in a 10‐μl volume mix containing 0.2‐μl Power SYBR Green PCR Master Mix, 5 pmol/L specific primers, and approximately 50 ng cDNA. The cycling parameters were 95°C for 10 min, followed by 45 cycles of 95°C for 20 s, 55°C for 20 s, and 72°C for 20 s. Threshold cycles and dissociation curves were determined with QuantStudio^™^ 6 Flex software, to confirm that only one PCR product was amplified and detected. Gene expression levels were normalized to 16S RNA. The Relative Expression Software Tool (version 2, REST 2008) was used to calculate the relative expression of genes in RT‐qPCR using the Pair Wise Fixed Reallocation Randomization Test (Pfaffl, Horgan, & Dempfle, 2002). The mathematical model used was based on the mean crossing point deviation between the sample and the control group, normalized by the mean crossing point deviation of the reference genes. Specific amplification efficiencies were included in the correction of the quantification ratio. Significant differences between groups were determined by ANOVA followed by the Tukey's LSD. The primers are listed in Table S3.

### 2.6 Mucus preparation

Healthy *Pseudosciaene crocea* were obtained from marine cultured cages in Ningde of Fujian Province, China. Skin mucus was prepared according to the method of Kong et al. (2015). After washing with sterile PBS (0.01 mol/L, pH = 7.2), the surface gel layer of the skin was scraped to collect skin mucus with a plastic spatula. This layer was homogenized in PBS, and the homogenate was centrifuged twice (20,000 × g, 4°C, 30 min) to remove particulate materials and then filtered through 0.45‐ and 0.22‐μm pore size filters, respectively. The mucus samples were adjusted to 1 mg protein/ml using the method described by Bradford (1976).

The animal experiment in this study was conducted in strict accordance with the recommendations outlined in the Guide for the Care and Use of Laboratory Animals of the National Institutes of Health. The protocol was approved by the Committee on the Ethics of Animal Experiments of the Animal Ethics Committee of Xiamen University (Acceptance No. XMULAC20120030).

### 2.7 In vitro adhesion assay

The bacterial adhesion was assayed as the method described by Huang, Hu, et al. (2016). Fifty microliter of mucus was evenly spread onto a 22 × 22‐mm glass slide area. After fixing with methanol for 20 min, 1 ml of bacterial suspension (10^8^ CFU/ml) was added onto the mucus‐coated glass slides. After incubating at 25°C for 2 hr in a humidified chamber, the slides were washed five times with PBS to remove nonadhering bacterial cells. Finally, the adhering bacterial cells were fixed with 4% methanol for 30 min, dyed with crystal violet for 3 min, and observed under a microscope (×1,000). The average number of bacteria adhering to a field of view of the glass surface was then determined. For each assay, 20 fields of view were counted and the average value was calculated.

### 2.8 Growth curves in the presence of glutathione (GSH)

*V. alginolyticus* was incubated at 28°C to the initial exponential growth phase (OD_600 nm_ = 0.2), and then GSH (Sigma‐Aldrich Co. LLC, St. Louis, MO) was added at concentrations of 10 mM according to Green et al. (2000). The controls received no treatment with GSH. The values of OD_600 nm_ were recorded at 0, 50, 100, 150, 200, 250, 300, 1,440, 2,880, and 4,320 min after the addition of GSH. From the OD_600 nm_ data, growth curves were plotted comparing the wild‐type and mutant strains in the presence and absence of the toxic substrate GSH. Three independent biological replicates were performed for each data point.

### 2.9 Hemolysis assay

Hemolysis assays were carried out as described by Tsou and Zhu (2010). Rabbit blood (Ping Rui Biotechnology Co. Ltd. Beijing, China) was washed three times with PBS. Five microliter of washed rabbit blood was mixed with 245 μl of culture supernatants and incubated at 37°C for 1 hr with shaking (220 r.p.m.). After incubation, samples were centrifuged, and the released hemoglobin was measured by OD_540 nm_. The percentage of total hemolysis was calculated by comparing the OD_540 nm_ of the samples with positive (100% lysis by 1% Triton X‐100) and negative controls. Three independent biological replicates were performed for each data point.

### 2.10 Biofilm assay

The biofilm assay for *V. alginolyticus* was performed as described by Luo et al. (2016). *V. alginolyticus* strains were overnight grown at 28°C in LB and then adjusted OD_600 nm_ to 0.2. Fifty microliter of bacterial culture was mixed with 150 μl of LB per well of a 96‐well plate and then incubated at 28°C for 24 hr. Then, wells were rinsed three times with sterile PBS, incubated (15 min) with 200 μl crystal violet (1%), washed with sterile PBS, and air dried. The stained biofilm was solubilized with 200 μl 33% acetic acid and then quantitated by measuring OD_590 nm_, six independent biological replicates (six technical replicates within each) were performed for each data point.

### 2.11 Artificial infection

It has been found by many laboratories that yellow croaker could not be cultured in the laboratory, while *V. alginolyticus* has been proven to be pathogenic to grouper (Gu et al., 2016). We also found that *V. alginolyticus* was pathogenic to *Epinephelus coioides* and led to similar symptoms. Therefore, *E. coioides* was used in this study for artificial infection. A total of 140 healthy individuals of *E. coioides* were randomly divided into seven groups. Twenty fish in each group were challenged with wild‐type and five stable silenced strains of *V. alginolyticus*, respectively. Each fish was intraperitoneal injected with 0.1‐ml bacterial suspension (1 × 10^7^ CFU/ml), while PBS instead of the bacterial suspension was used as the negative control. The mortality was recorded daily for 15 days.

### 2.12 Data processing

The results are presented as means ± *SE*. The data were statistically analyzed with one‐way ANOVA followed by Dunnett's multiple comparison test using SPSS 13.0 software (IBM Corporation, Armonk, NY, USA). A value of *P *<* *.05 indicated a significant difference.

## 3 RESULTS

### 3.1 Effects of different environmental conditions on the expression of *oppABCDF*

**Figure 1:**
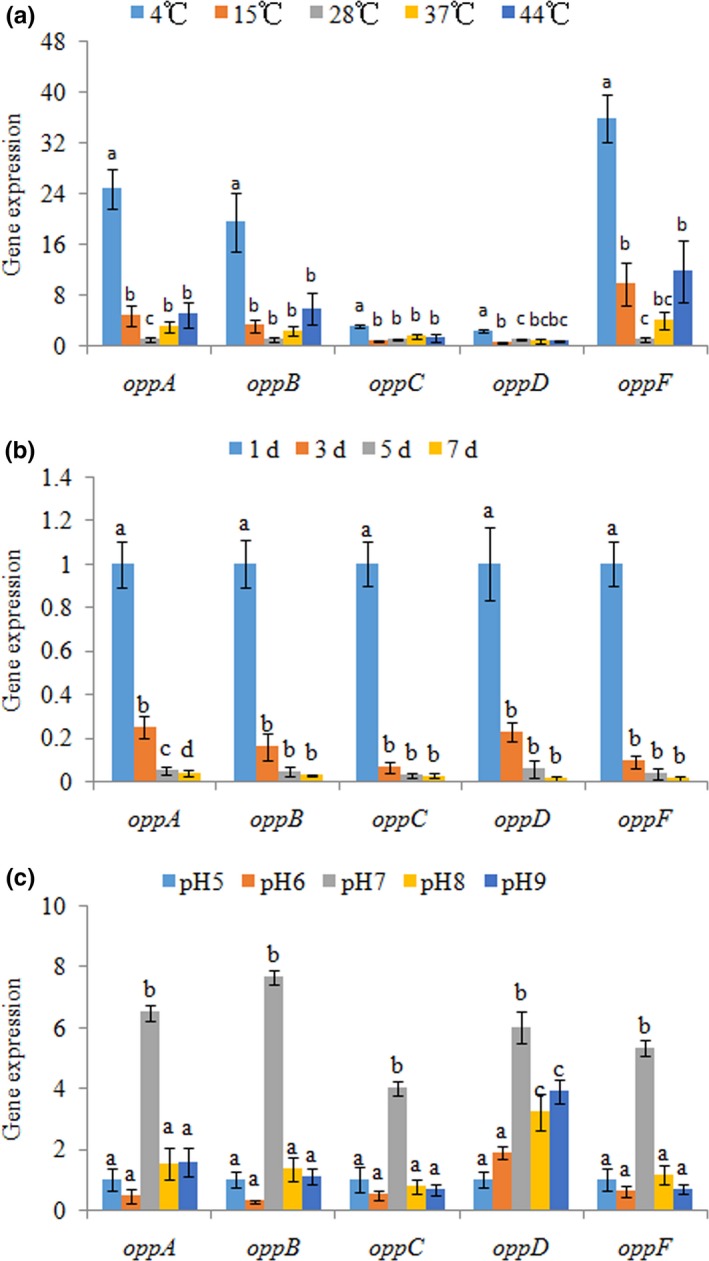
RT‐qPCR analysis of the expression of *oppABCDF* in the *Vibrio alginolyticus* under different temperatures (a), different starvation time (b), and various pH values (c). The data are presented as the means ± *SD*, each treatment consisted of six independent biological replicates. The means of treatments not sharing a common letter are significantly different at *P *< .05

The expression levels of *oppABCDF* displayed a similar inverted U‐shaped trend (Figure 1a). The highest expression was observed at 4°C. Based on the results, low temperatures apparently had a greater impact than high temperatures, and the *oppF* gene appeared to be the most sensitive to low temperature, while *oppD* appeared to be the least sensitive to low temperature.

Starvation significantly reduced gene expression in a time‐dependent manner (Figure 1b). After 3 days of treatment, the effect of starvation on *oppC* was the strongest, while the effect of starvation on *oppA* was the weakest. Furthermore, starvation for 7 days significantly downregulated expression of *oppA*,* oppB*,* oppC*,* oppD,* and *oppF*.

The expression levels of *oppABCDF* displayed a similar inverted U‐shaped trend (Figure 1c). The highest expression was observed at pH = 7.0. The *oppB* gene appeared to be the most sensitive to different pH, whereas *oppD* appeared to be the least sensitive.

### 3.2 Effects of transient gene silencing on adhesion

**Figure 2:**
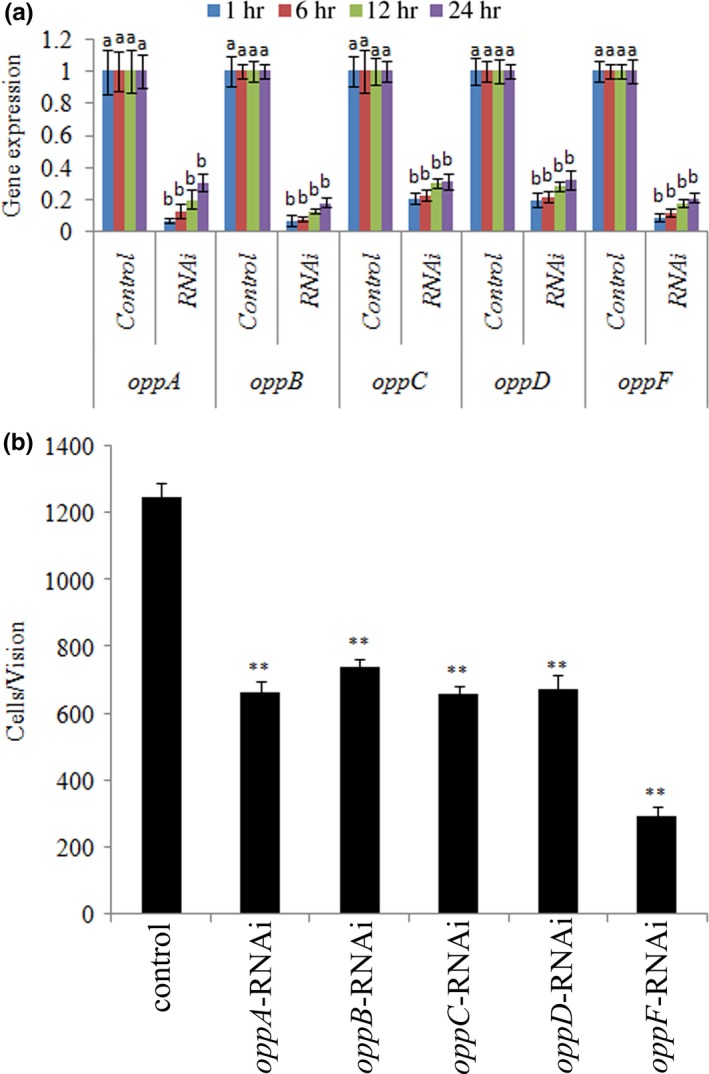
Transient gene silencing reduced the adhesion of *Vibrio alginolyticus*. (a) RT‐qPCR analysis of the expression of *oppABCDF* after transient gene silencing at 1, 6, 12, and 24 hr compared to the control. The data are presented as the means ± *SD*, each treatment consisted of six independent biological replicates. The means of treatments not sharing a common letter are significantly different at *P *< .05. (b) The adhesion capacity to mucus of transient silenced *V. alginolyticus* at 2 hr. The data are presented as the means ± *SD*, three independent biological replicates were performed for each data point. **P < .01 compared with control subjects

*V. alginolyticus* treated with scrambled siRNA is used as a control here (Figure 2). Compared to the control, the expression levels of these target genes were significantly reduced at 1, 6, 12, and 24 hr after *V. alginolyticus* was treated with siRNAs (Figure 2a). At 1, 6, 12, and 24 hr after transient gene silencing, *oppABCDF* was significantly reduced. Reductions in target gene expression indicated the successful application of these siRNAs.

**Figure 3:**
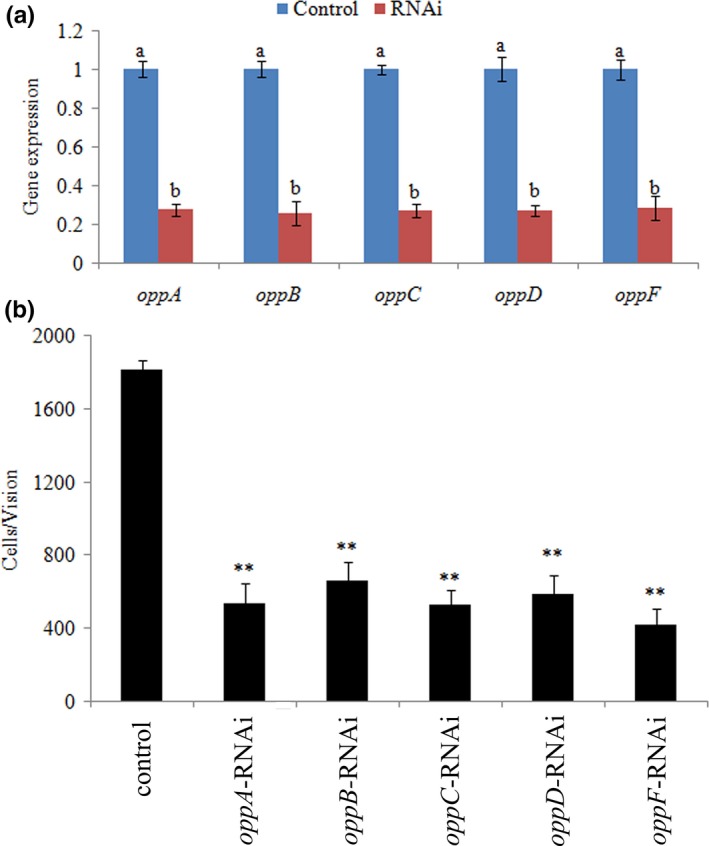
Stable gene silencing reduced the adhesion of *Vibrio alginolyticus*. (a) RT‐qPCR analysis of the expression of *oppABCDF* after stable gene silencing compared to the control. The data are presented as the means ± *SD*, six independent biological replicates were performed for each data point. The means of treatments not sharing a common letter are significantly different at *P *< .05. (b) The adhesion capacity of stable silenced *V. alginolyticus* to mucus. The data are presented as the means ± *SD*, three independent biological replicates were performed for each data point. **P < .01 compared with control subjects

As *V. alginolyticus* with RNAi treatments displayed significant silencing at 1–6 hr, the in vitro adhesion assay was performed after transient gene silencing for 2 hr. The results of the in vitro adhesion assay indicated significantly decreased *V. alginolyticus* adhesion ability under RNAi conditions (Figure 2b). After transient gene silencing for 2 hr, the adhesion ability of *V. alginolyticus* treated with *oppA‐*,* oppB‐*,* oppC‐*,* oppD‐,* and *oppF*‐RNAi was significantly reduced.

### 3.3 Effects of stable gene silencing on adhesion

As shown in Figure 3a, the expression levels of *oppA*,* oppB*,* oppC*,* oppD,* and *oppF* were significantly reduced in stably silenced clones.

**Figure 4:**
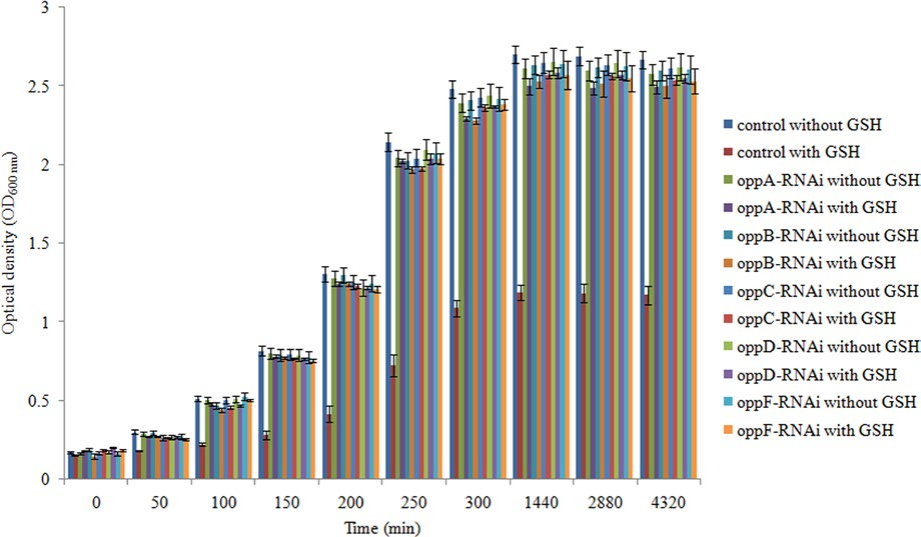
Growth of wild‐type and stable silenced strains in the presence or absence of glutathione (GSH; *n* = 3)

The adhesion ability of the stably silenced clones was detected, which showed that the numbers of adherent bacteria of the control group were approximately 1,811 ± 50 cells/view, whereas the corresponding numbers of adherent bacteria of the *oppA‐*,* oppB‐*,* oppC‐*,* oppD‐,* and *oppF*‐RNAi groups were 540 ± 110, 660 ± 101, 530 ± 80, 590 ± 101, and 420 ± 91 cells/view, respectively (Figure 3b). This demonstrated that the adhesion ability of *V. alginolyticus* was significantly impaired after stable gene silencing.

### 3.4 Effects of stable gene silencing on peptide internalization

GSH was used as a toxic substrate to investigate the abilities of peptides capture of the *oppA‐*,* oppB‐*,* oppC‐*,* oppD‐,* and *oppF*‐RNAi strains, and the results are shown in Figure 4. The wild‐type strain showed lower growth profile in the presence of the toxic substrate than in the absence of the toxic substrate, while the stable silenced strains showed the same growth profile in the presence and absence of the toxic substrate (Figure 4).

**Figure 5:**
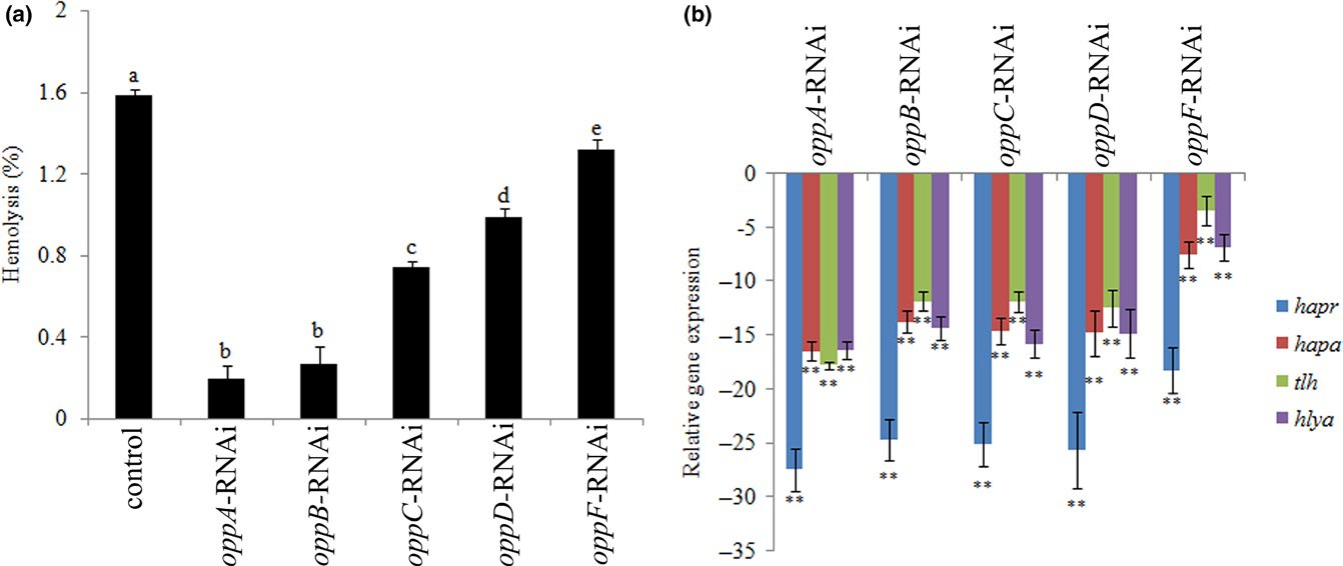
Stable gene silencing reduced the hemolytic activity of *Vibrio alginolyticus*. (a) The hemolytic activity of *V. alginolyticus* strains after stable gene silencing. The data are presented as the means ± *SD*, three independent biological replicates were performed for each data point. The means of treatments not sharing a common letter are significantly different at *P *< .05. (b) RT‐qPCR analysis of the expression of *hapr*,* hapa*,* tlh,* and *hlya* after stable gene silencing compared to the control. The data are presented as the means ± *SD*, six independent biological replicates were performed for each data point. **P < .01 compared with control subjects

### 3.5 Effects of stable gene silencing on hemolytic activity

The hemolytic activity was significantly decreased in *oppA‐*,* oppB‐*,* oppC‐*,* oppD‐,* and *oppF*‐RNAi strains compared to the wild‐type strain. The suppression of hemolytic activity was highest in the *oppA*‐RNAi strain and lowest in the *oppF*‐RNAi strain (Figure 5a).

**Figure 6:**
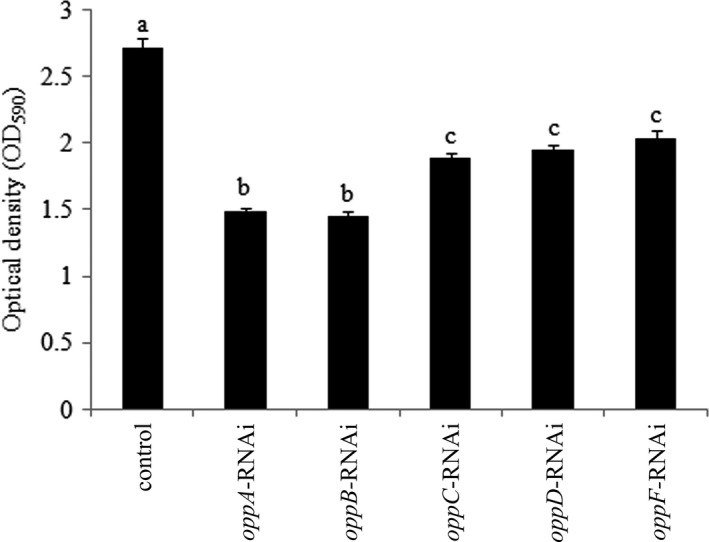
Biofilm formation of stable silenced *Vibrio alginolyticus* strains. OD_590_ of stained biofilm in the colony of each strain. The data are presented as the means ± *SD*, six independent biological replicates (six technical replicates within each) were performed for each data point. The means of treatments not sharing a common letter are significantly different at *P *< .05

*hapr*,* hapa*,* tlh,* and *hlya* are important genes closely related to the hemolytic activity of Vibrios, and their expression levels were detected in stably silenced *V. alginolyticus* (Figure 5b). Compared to the control group, the levels of *hapr*,* hapa*,* tlh,* and *hlya* expression were significantly reduced in all five RNAi groups. After stable gene silencing, *oppA‐*RNAi exhibited the strongest repression of these hemolytic genes, while *oppF‐*RNAi exhibited the weakest repression of these hemolytic genes. Meanwhile, after stable gene silencing*,* expression of *hapr* presented the greatest decreases.

### 3.6 Effects of stable gene silencing on biofilm production

The ability to form biofilms was significantly decreased in *oppA‐*,* oppB‐*,* oppC‐*,* oppD‐,* and *oppF*‐RNAi strains compared to the wild‐type strain, while *oppA*‐ and *oppB*‐RNAi was more effective than any other strains (*P *<* *.05) (Figure 6). However, *oppF*‐RNAi displayed less effective than other strains.

**Figure 7:**
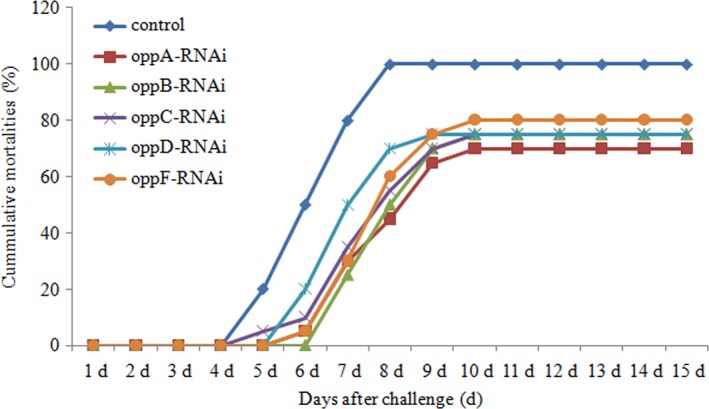
The cumulative mortality of *Epinephelus coioides* injected with wild‐type and *oppA‐*,* oppB‐*,* oppC‐*,* oppD‐,* and *oppF*‐RNAi strains during 15 days postchallenge

### 3.7 Effects of stable gene silencing on virulence

Monitoring the fish mortality for postchallenge revealed that mortality was remarkably lower in the groups treated with *oppA*‐, *oppB*‐, *oppC*‐, *oppD*‐, and *oppF*‐RNAi strains compared to the control group, Figure 7. The relative percent of survival was 0%, 30%, 25%, 25%, 25%, and 20% in fish challenged with wild‐type and *oppA*‐, *oppB*‐, *oppC*‐, *oppD*‐, and *oppF*‐RNAi strains, respectively. The group challenged with wild‐type and *oppC*‐RNAi strain started to die since day 5, then the group challenged with *oppA*‐, *oppD*‐, and *oppF*‐RNAi strains started to die since day 6. The group challenged with *oppB*‐RNAi strains did not die until day 7. At day 5, the death ratio caused by wild‐type was five times higher than the death ratio caused by *oppC*‐RNAi strain.

## 4 DISCUSSION

In order to verify the role of *oppABCDF* in the regulation of *V. alginolyticus* virulence, gene silencing was performed in this study. After gene silencing, the expression level of *oppABCDF* was significantly reduced. Meanwhile, GSH, as a toxic substrate proved to be an effective tool in the characterization of mutant strains for the Opp peptide transporter in *Mycobacterium bovis* and *C. pseudotuberculosis* (Green et al., 2000; Moraes et al., 2014), was used to investigate the abilities of peptides capture after stable gene silencing. Our results showed that the wild‐type strain was sensitive to 10 mM of GSH, while the stable silenced strains were resistant to the toxic effects of GSH at this concentration. These results indicated that the function of the Opp system to capture peptides from the extracellular environment was impaired after stable gene silencing. Meanwhile, until 72 hr, there was still no significant difference between control and the silenced strains grown without GSH, which meant the deficient of Opp system did not affect the growth of *V. alginolyticus* significantly. Therefore, we think the phenotypic changes in silenced strains were mainly due to the missing gene activity instead of the decreased general fitness of the silenced strains.

The pathogenic process of bacteria is generally divided into adhesion, invasion, colonization, proliferation, and production of toxins (Huang et al., 2015). According to our results, *oppABCDF* contributed in multistep of *V. alginolyticus* pathogenesis.

Bacterial adhesion to host surfaces is one of the initial steps in the infection process (Pizarro‐Cerda & Cossart, 2006). Host mucus is abundantly found on the surface of the skin, gills, and gut lining; therefore, it is the first site of interaction between the pathogen and its host (Chen, Yan, Wang, Zhuang, & Wang, 2008). Samen et al. (2004) found that deletion of the *oppB* gene reduced the adherence of *Streptococcus pneumoniae* to epithelial cells by 26%, impaired its adherence to fibrinogen and fibronectin by 42% and 33%, respectively. However, *oppA*‐, *oppC*‐, *oppD*‐, and *oppF* were not proven to be associated with adhesion regulation. Meanwhile, no previous study has concerned about the relationship between bacterial adhesion to mucus and *oppABCDF* expression. In this study, RNAi‐mediated silencing of these genes reduced bacterial adhesion to mucus. For the first time, these results demonstrate that *oppABCDF* plays a key role in *V*. *alginolyticus* adhesion to mucus.

After adhesion to its host, bacteria are possible to start the invasion, besides they should try to protect themselves against the host immune system during this process. Therefore, various ways were developed, such as biofilm production (Atwood et al., 2015; Rybtke et al., 2015; She et al., 2016). Lee et al. (2004) found that the *oppA* is involved in biofilm production of *Vibrio fluvialis*. However, whether *oppBCDF* was also involved in biofilm production of *Vibrio fluvialis* was not verified (Lee et al., 2004). In this study, results showed that the stably silenced strains can substantially reduce biofilm production compared with the wild‐type strain. While *oppA*‐ and *oppB*‐RNAi were more effective than any other strains, *oppF*‐RNAi displayed less effectiveness than other strains. These results demonstrated that *oppABCDF* play an important role in *V. alginolyticus* biofilm production, while *oppA* and *oppB* has the greatest impact and *oppF* has the least.

Hemolysin is an important virulence factor in the pathogenesis of many *Vibrio* species (Syed et al., 2009). Wu et al. (2007) found that *oppA* is essential for the hemolytic activity of *Vibrio furnissii* in vitro, while the function of *oppBCDF* in the regulation of hemolytic activity was not verified. In this study, *oppABCDF* was stable silenced in *V. alginolyticus* and the hemolytic activity was detected. According to the results, the hemolytic activity was significantly decreased in *oppA*‐, *oppB*‐, *oppC*‐, *oppD*‐, and *oppF*‐RNAi strains compared to the wild‐type strain, and *oppA*‐RNAi was the most effective than any other strains, while *oppF*‐RNAi was the least effective. In addition, expression levels of *hapr*,* hapa*,* tlh,* and *hlya*, which are important genes closely related to the hemolytic activity of *Vibrio* (Syed et al., 2009), were also detected in stably silenced *V. alginolyticus*. Compared to the control group, the levels of *hapr*,* hapa*,* tlh,* and *hlya* expression were significantly reduced in all five RNAi groups, suggesting that the expression of these genes is affected by *oppA*‐, *oppB*‐, *oppC*‐, *oppD*‐, and *oppF*‐RNAi. After stable gene silencing, *oppA‐*RNAi exhibited the strongest repression of these hemolytic genes, while *oppF‐*RNAi exhibited the weakest repression of these hemolytic genes. This might be an explanation for the lowest hemolytic activity in *oppA*‐RNAi strain and the highest hemolytic activity in *oppF*‐RNAi strain. Meanwhile, expression level of *hapr* was decreased most significantly in all stable gene silenced strains, suggesting that the silencing of *oppABCDF* had the greatest impact on *hapr*.

Several studies performed on pathogenic bacteria of the genera *Staphylococcus* sp., *Streptococcus* sp., and *Mycobacterium* sp. have shown that Opp mutant strains show reduced virulence (Coulter et al., 1998; Sassetti & Rubin, 2003; Wang et al., 2005). Gominet et al. (2001) and Slamti and Lereclus (2002) demonstrated that the Opp systems could also activate a pleiotropic virulence regulon in *Bacillus thuringiensis*. In this study, the effect of *oppABCDF* on the virulence of *V. alginolyticus* was also evaluated. Monitoring the fish mortality for postchallenge revealed that mortality was remarkably lower in the groups treated with *oppA‐*,* oppB‐*,* oppC‐*,* oppD‐,* and *oppF*‐RNAi strains compared to the control group. Meanwhile, groups treated with *oppABCDF*‐RNAi strains started to die later than the control group. These indicated that the silencing of *oppABCDF* had significant impact on virulence of *V. alginolyticus*. Furthermore, after stable gene silencing, *oppA‐*RNAi exhibited the strongest reduction in virulence, while *oppF‐*RNAi exhibited the weakest reduction in virulence, which was in accordance with the trends of hemolytic activity, expression levels of hemolytic genes, and production of biofilm. Therefore, the reduction in virulence after gene silencing might be a result of combined effect of decreased hemolytic activity and production of biofilm.

Many pathogenic bacteria can induce an adaptable response to environmental stimuli, primarily by altering gene expression (Beier & Gross, 2006). Environmental changes have also been shown to influence *opp* expression. For example, transcription of *oppA* genes is induced by a temperature downshift in *L. monocytogenes* (Borezee, Pellegrini, & Berche, 2000) and *Bacillus subtilis* (Budde, Steil, Scharf, Volker, & Bremer, 2006); expression of the *E. coli opp* operon is upregulated under anaerobic conditions (Andrews & Short, 1986). In our present study, the expression levels of *oppABCDF* were found to be sensitive to different temperatures, changes in pH, and increased starvation time. On the other hand, Yan et al. (2007) proved that *V. alginolyticus* adhesion was remarkably influenced by those environmental factors, which suggested that the changes in expression of *oppABCDF* may be an important factor influencing adhesion under those environmental conditions. On the other hand, our results showed an induction of *oppABCDF* expression at low temperature, which was in accordance with previous report from Borezee et al. (2000) that *oppA* is required for bacterial growth at low temperature. Both of them further strengthened the necessity of this study.

In conclusion, our results indicated that (1) *oppABCDF* contributed in multistep of *V. alginolyticus* pathogenesis, including adhesion, biofilm production, and hemolytic activity; (2) *oppABCDF* was sensitive to different temperatures, changes in pH, and increased starvation time.
