# Supplementary material for: Contributions of the oligopeptide permeases in multistep of Vibrio alginolyticus pathogenesis
Source: Microbiologyopen. 2017 Jul 17;6(5):e00511. doi: 10.1002/mbo3.511 (PMC5635161; doi:10.1002/mbo3.511)
Supplement: Supplementary file 1 [file MBO3-6-na-s001.doc]

**Table S1 siRNA Sequence**

| **Target gene** | **siRNA for transient gene silence** |
| --- | --- |
| *oppA* | F: 5' GCUGGCAACGCUUACUAAATT 3'  R: 5' UUUAGUAAGCGUUGCCAGCTT 3' |
| *oppB* | F: 5' GCCUAUGCCAGAAGUGGUATT 3'  R: 5' UACCACUUCUGGCAUAGGCTT 3' |
| *oppC* | F: 5' GCGUAAUGAUGCGCAUUAUTT 3'  R: 5' AUAAUGCGCAUCAUUACGCTT 3' |
| *oppD* | F: 5' GCGUCAGCGAAUCAUGAUATT 3'  R: 5' UAUCAUGAUUCGCUGACGCTT 3' |
| *oppF* | F: 5' CCAUACAAGCUCAGGUCAUTT 3'  R: 5' AUGACCUGAGCUUGUAUGGTT 3' |
| Negative control | F: 5'-UUCUCCGAACGUGUCACGUTT-3'  R: 5'-ACGUGACACGUUCGGAGAATT-3' |
