# Supplementary material for: Contributions of the oligopeptide permeases in multistep of Vibrio alginolyticus pathogenesis
Source: Microbiologyopen. 2017 Jul 17;6(5):e00511. doi: 10.1002/mbo3.511 (PMC5635161; doi:10.1002/mbo3.511)
Supplement: Supplementary file 2 [file MBO3-6-na-s002.doc]

**Table S2 Oligonucleotides used in producing shRNA for stable gene silencing**

| **Target** | **shRNA sequence for** **stable gene silence** |
| --- | --- |
| *oppA* | F:5'-GATCCGCTGGCAACGCTTACTAAATTTTCAAGAGAAATTTAGTAAGCGTTGCCAGCTTTTTTGCATG-3'  R:5'-GCGACCGTTGCGAATGATTTAAAAGTTCTCTTTAAATCATTCGCAACGGTCGAAAAAAC-3' |
| *oppB* | F:5'-GATCCGCCTATGCCAGAAGTGGTATTTTCAAGAGAAAAACGGCAAATGCTTTCTCCTTTTTTGCATG-3'  R:5'-GCGGATACGGTCTTCACCATAAAAGTTCTCTTTTTGCCGTTTACGAAAGAGGAAAAAAC-3' |
| *oppC* | F:5'-GATCCGCGTAATGATGCGCATTATTTTTCAAGAGAAAATAATGCGCATCATTACGCTTTTTTGCATG-3'  R:5'-GCGCATTACTACGCGTAATAAAAAGTTCTCTTTTATTACGCGTAGTAATGCGAAAAAAC-3' |
| *oppD* | F:5'-GATCCGCGTCAGCGAATCATGATATTTTCAAGAGAAATATCATGATTCGCTGACGCTTTTTTGCATG-3'  R:5'-GCGCAGTCGCTTAGTACTATAAAAGTTCTCTTTATAGTACTAAGCGACTGCGAAAAAAC-3' |
| *oppF* | F:5'-GATCCCCATACAAGCTCAGGTCATTTTTCAAGAGAAAATGACCTGAGCTTGTATGGTTTTTTGCATG-3'  R:5'-GGGTATGTTCGAGTCCAGTAAAAAGTTCTCTTTTACTGGACTCGAACATACCAAAAAAC-3' |
