# Supplementary material for: Contributions of the oligopeptide permeases in multistep of Vibrio alginolyticus pathogenesis
Source: Microbiologyopen. 2017 Jul 17;6(5):e00511. doi: 10.1002/mbo3.511 (PMC5635161; doi:10.1002/mbo3.511)
Supplement: Supplementary file 3 [file MBO3-6-na-s003.doc]

**Table S3 Primers for q RT-PCR**

| **Gene** | **Primers for qRT-PCR** |
| --- | --- |
| *oppA* | F: 5' AAGCAAGCCGCAGAGC 3'  R: 5' AATGGCCGCAAACCAA 3' |
| *oppB* | F:5' ATTGCGGCATTGAACC 3'  R: 5' TGAGCACGGGAAGAACA 3' |
| *oppC* | F: 5' TGTTCTGCCTAATGTGCTT 3'  R: 5' CGGTCTTTCGGGTCAA 3' |
| *oppD* | F: 5' TTGACGCTATTACGAGACTTAC 3'  R: 5' TGGGTTACCTGGGATGG 3' |
| *oppF* | F: 5' GCGTTATGGTGATGTATCTTG 3'  R: 5' CTTCCGTGGCTTCTGG 3' |
| *hapr* | F: 5' ATGCAATGATCGAGCTTGTC 3'  R: 5' TGTTCTGAATCGTGCTGGTC 3' |
| *hapa* | F: 5' CGTGGCGGCGAATGATGTA 3'  R: 5' GGGTCCCGATGTGGTAACAATAA 3' |
| *hlya* | F: 5' AGCAGCGTGTGGGACAAGAT 3'  R: 5' AGGTTGCCGTGATGGATTTC 3' |
| *tlh* | F: 5' CACTTGGGAGTGGGCAAAGA 3'  R: 5' TGGTTGACACCAAGCGTCTCT 3' |
